# DNA demethylation enhances myoblasts hypertrophy during the late phase of myogenesis activating the IGF-I pathway

**DOI:** 10.1007/s12020-013-0142-5

**Published:** 2013-12-24

**Authors:** Pamela Senesi, Livio Luzi, Anna Montesano, Ileana Terruzzi

**Affiliations:** 1 Department of Biomedical Sciences for Health, University of Milan, Milan, Italy; 2 Metabolism Research Centre and Department of Endocrinology and Metabolic Diseases, San Donato Hospital and Scientific Institute, Milan, Italy; 3 Division of Metabolic and Cardiovascular Science, Metabolism, Nutrigenomics and Cellular Differentiation Unit, San Raffaele Scientific Institute, Milan, Italy

**Keywords:** Muscle differentiation, DNA methylation, 5-Azacytidine, Hypertrophic process, C2C12 myoblasts, IGF-I

## Abstract

Skeletal muscle regeneration and hypertrophy are important adaptive responses to both physical activity and pathological stimuli. This research was performed to investigate DNA demethylation action on the late phase of muscle differentiation and early stage of hypertrophy. The epigenetic process involved in myogenesis was studied with the DNA-demethylating agent 5-azacytidine (AZA). We induced muscle differentiation in C2C12 mouse myoblasts in the presence of 5 μM AZA and growth or differentiation medium for 48, 72, and 96 h. To study a potential AZA hypertrophic effect, we stimulated 72 h differentiated myotubes with AZA for 24 h. Unstimulated cells were used as control. By western blot and immunofluorescence analysis, we examined AZA action on myogenic regulatory factors expression, hypertrophic signaling pathway and myotube morphology. During differentiation, protein levels of myogenic markers, Myf6 and Myosin Heavy Chain (MyHC), were higher in AZA stimulated cells compared to control. Myostatin and p21 analysis revealed morphological changes which reflect a tendency to hypertrophy in myotubes. In AZA stimulated neo formed myotubes, we observed that IGF-I pathway, kinases p70 S6, 4E-BP1, and ERK1/2 were activated. Furthermore, AZA treatment increased MyHC protein content in stimulated neo myotubes. Our work demonstrates that DNA demethylation could plays an important role in promoting the late phase of myogenesis, activating endocellular pathways involved in protein increment and stimulating the hypertrophic process.

## Introduction

Skeletal muscle regeneration and hypertrophy are important adaptive responses to both disease and physical activity [1]. Muscle repair is controlled by satellite cells (SC), representing skeletal muscle stem cells [2]. In response to injury, quiescent SC start to proliferate generating myogenic precursor cells (myoblasts). Myoblasts withdraw from the cell cycle and form myotubes [3].

This process is principally governed by muscle regulatory factors (MRFs), including MyoD and Myf6 [4, 5]. MRFs regulate the cellular cycle progression, in particular Myf6 cooperates with p21, a cell cycle regulator protein, to promote muscle specific protein synthesis [6] such as Myosin Heavy Chain (MyHC). Instead, the end of myogenesis [7] is lead by Myostatin (Mnst). Mstn, a member of the transforming growth factor-β superfamily, is an important negative regulator of skeletal muscle development. Naturally, Mnst occurring mutations, as well as experimental knockout of the *Mstn* gene, lead to hypermuscular phenotype [8]. In vitro model of skeletal muscle cells, Mstn is predominantly localized in the nuclei of differentiated, polynucleated myotubes and down regulates the muscle genes expression [9].

Muscle hypertrophy is an increment of existing muscle fibers size [10], associated with an enhanced protein accumulation. Insulin growth factor I (IGF-I) are crucially involved in hypertrophic process induced by Growth Hormone (GH) treatment or exercise [11, 12]. In recent years, Spangerburg and other investigators have tried to understand the mechanisms by which IGF-I may mediate changes in muscle mass during mechanical loading. [13–15]. The data, obtained by these researchers, are in part controversial [16–19]: inhibition of IGF-I activity does not eliminate increased muscle mass through mechanical load, but driving IGF-I enhances the effects of load. Although a controversial point of view, there is no doubt that IGF-I could play a central role in muscle hypertrophy and adaptation. In fact, IGF-I overexpression is sufficient to induce muscle hypertrophy, modulating the entire circuit necessary to guarantee it: an increase in protein synthesis, a decrease in protein degradation, an activation and a fusion of satellite cells [20–22].

Insulin growth factor I binding activates the IGF-I receptor (IGF-I R), a receptor tyrosine kinase. The IGF-I R subsequently recruits the insulin receptor substrate (IRS-1), which results in the activation of two signaling pathways: the mitogen-activated protein kinases (MAPK) pathway and the phosphatidylinositol 3-kinase (PI-3 K) pathway [23, 24].

The MAPK pathway is crucial in mitosis-competent cells for cell proliferation and survival [25]. Extracellular signal regulated kinases (ERK1 and ERK2), members of the MAPK family, are involved in the regulation of muscle mass. Myoblasts/myotubes have a unique biphasic requirement for ERK activity [26]. ERK1/2 are critical for growth factor-induced cellular proliferation, inhibitory to myoblastic differentiation. These kinases are required for myotube fusion and appear critical to this last process [27]. Moreover, in adult skeletal muscle, high-intensity exercises have been shown to activate the MAPK–ERK pathway and in vivo studies showed that MAPK-dependent pathways affect both fiber size and fiber type [28].

The PI-3 K pathway is the predominant pathway that stimulates muscle protein synthesis and is believed to be required for a normal hypertrophic response. PI-3 K direct target is AKT [10, 24]. Under normal conditions, AKT activation results in the formation of a signaling complex termed TORC1, an important component of which is mTOR [29]. Activation of mTOR leads to phosphorylation of ribosomal protein S6 Kinase (p70 S6 K). p70 S6 K phosphorylates an important ribosomal subunit that is necessary for muscle protein translation, and deletion of p70 S6 K in muscle results in smaller muscle fibers [30]. Furthermore, mTOR directly phosphorylates the eukaryotic initiation factor 4E (4E-BP1). Once phosphorylated, 4E-BP1 releases its inhibitory effect on the translation initiation factor elF4E, which impairs inhibition of translation initiation by coupling with the end CAP of mRNA [30, 32].

Recently, discoveries in the field of skeletal muscle biology have made an effort to understand how epigenetic modifications affect the myogenic lineage acquisition [33].

The observation that treatment with a methyltransferase inhibitor, 5-azacytidine (AZA), converts C3H10T1/2 embryonic fibroblasts into muscle cells providing the first relationship between DNA methylation and myogenesis [34]. This correlation is further underscored by the finding that promoters of MRFs, MyoD and Myogenin, are demethylated during muscle cell differentiation [35, 36]. However, the specific impact of DNA demethylation on late phase of differentiation and on muscle mass regulation is not completely understood.

In our study, we used AZA to induce DNA demethylation during differentiation stages of C2C12 cell line, an established model of satellite cell growth and differentiation [37, 38]. Our data show that DNA demethylation could stimulate myoblasts differentiation and promote hypertrophic process, through the activation of IGF-I pathway.

## Materials and methods

### Materials

Anti calnexin (H-70), anti MyoD (C-20), anti Myf6 (C-19), anti Myostatin (GDF-8: N-19), anti MyHC (H-300), anti p21 (C-19), anti IGF-I receptor-β (C-20), anti phosho p70 S6-kinase (Thr421/Ser 424), anti p70 S6-Kinase (C-18), anti 4E-BP1 (R-113), anti eIF-4E (P-2), anti p-ERK1/2 (E-4), anti-ERK1 (K-23), and anti ERK2 (C-14) primary antibodies and peroxidase or rhodamine-conjugated secondary antibodies were purchased from Santa Cruz Biotechnology (Santa Cruz, CA, USA). All other reagents were purchased from Sigma Chem. Co. (St. Louis, MO, USA). Mouse C2C12 myoblastic cells were purchased from European Collection of Animal Cell Cultures (ECACC).

### Experimental protocol

C2C12 myoblasts were cultured at 37 °C in humidified 5 % CO_2_ atmosphere in growth medium (GM), containing DMEM (Dulbecco Modified Eagle Medium) supplemented with 20 % (v/v) FBS (fetal bovine serum), 1 % penicillin–streptomycin, and 1 % l-glutamine. Differentiation process is initiated upon reaching subconfluence by switching C2C12 myoblasts in differentiation medium (DM), containing DMEM supplemented with 1 % horse serum, antibiotics, and 1 % l-glutamine [38]. While, C2C12 cells, maintained in GM, spontaneously fuse in neo myotubes as a result of the achievement of myoblasts confluence [39].

To study DNA demethylation action on differentiation process, cells, seeded at 6 × 10^2 ^cells/cm^2^, were grown until 80 % of confluence and then were maintained in GM or DM containing 5 μM AZA (GMAZA and DMAZA). 5 μM AZA represents the optimal concentration to stimulate cells without cytotoxic effects [34, 40]. This evidence was, furthermore, validated by our dose response experiments (data not shown). Control cells were maintained in GM or DM. Medium, with or without AZA, was changed every 24 h. Cells were analyzed during intermediate (48 h) and late (72 and 96 h) differentiation phases.

To investigate DNA demethylation effects on hypertrophic process, we used C2C12 cells after 72 h from differentiation induction in DM: in our model, most of the myotubes were completely formed in this phase. Neo formed myotubes were treated with 5 μM AZA for 24 h (AZAMT). Unstimulated cells (DMMT) were used as control.

### Growth curve and cell vitality test

C2C12 myoblasts were plated in 35 mm^2^ culture dishes and grown in presence of GMAZA or DM. Control cells were cultured in GM. Experiment continued until the control cells have reached subconfluence [41]. Every day, the cells were trypsinized and stained with trypan blue. Both viable (non-stained) and non-viable (blue) cells were counted using a hemacytometer. The total cell count average values for each single day were used to plot a growth curve for treated and controlled myoblasts. Cell vitality was calculated by dividing the non-stained viable cell count by the total cell count.

### Global DNA methylation assay

The total genomic DNA was extracted from the cells (treated with GM, DM, GMAZA or DMAZA) using the Qiagen DNA Mini Kit (Qiagen Sciences, Maryland, MD) following the manufacturer’s instructions. The integrity and purity of DNA were spectrophotometrically examined according to its A260/A280 absorption. The Global DNA methylation levels were determined using MethylFlash™ Methylated DNA Quantification Kit (Epigentek, NY, USA) according to the manufacturer’s instruction. The methylated fraction of DNA is recognized by a 5-methyl-cytosine (5-mC) antibody. With this colorimetric kit, the amount of methylated DNA, which is proportional to the optical density intensity, is quantified through an enzyme-linked immunosorbent assay-like reaction (ELISA assay). The reference DNA fragments containing 5-mC and 5-cytosine are used respectively as the positive standard and negative control to generate the standard curve. In particular, manufacturer’s instruction indicates that the percentage of 5-mC in the total DNA is calculated using the following formula:

$$\% \;5{\text{-mC}} = \frac{Sample\,OD - Negative\,control\,OD}{2 \times Slope \times Input \,DNA \,amount} \times \,100\;\%$$

Slope of the standard curve is determined using linear regression [42].

### Immunoblotting analysis

C2C12 cells were homogenized in lysis buffer (50 mM Tris/HCl, pH 7.4, 150 mM NaCl, 1 % Triton X-100, 1 mM sodium orthovanadate (Na_3_VO_4_), 1 mM EDTA, 1 mM PMSF, 1 mg/ml aprotinin, 1 mg/ml leupeptin, and 1 mg/ml pepstatin) and the obtained samples were sharked for 1 h at 4 °C. Detergent-insoluble material was removed from the cell suspension by centrifugation at 12,000×*g* for 30 min. Protein content was quantified using Bradford method. Aliquots of 30 μg supernatant proteins were resolved by SDS-PAGE and transferred to nitrocellulose membrane (Protran^®^ Whatman^®^ Schleicher & Schuell), as described [43]. The membranes were incubated with specific primary antibodies and then with HRP-conjugated secondary antibodies. Equal protein loading per sample was confirmed using antibody anti-calnexin. Immunoreactive bands were visualized by an enhanced chemiluminescence method (Amersham Pharmacia Biotech, Piscataway, NJ, USA). Densitometric analysis was performed using the Scion Image Software (Scion Corporation, Frederick, MD, USA).

### Immunofluorescence analysis and phase contrast microscopy

Cells were fixed in 4 % paraformaldehyde, and indirect immunofluorescence analysis was performed as described [43]. Cells were observed using fluorescence Leica DM IRE2 microscopy and Nikon Eclipse 50I microscopy. The images of myotubes were captured using respectively IM50 software and Nis-Elements D 4.00 software (Leica Microsystems, Switzerland and Nikon Instruments Europe BV, Netherlands). To verify that cells number in all conditions was superimposable, nuclei were revealed with DAPI staining. For myotube diameter and length dimensions, the average measurement was generated from approximately 150 myotubes. Ten fields were randomly chosen and all MyHC-positive multinucleated cells (at least 3 nuclei) in each field were measured. In detail, an average diameter per myotube was calculated as the mean of three measurements taken along the length of the myotube. As proposed by Menconi et al. [44], the measurements were conducted in a “blinded” fashion on coded pictures with the investigator being unaware of the group from which the cultures originated. The experiments were repeated three times. Live C2C12 cells were examined and images acquired by phase contrast microscopy using the same microscope and digital system described above.

### Statistical analysis

Data are converted to fold change (FC) relative to the control and presented as the mean ± SD. Statistical significances differences between treatments were analyzed using one-way ANOVA and Bonferroni post test or t-tests. Results were considered significant when *p* ≤ 0.05.

## Results

**Fig. 1:**
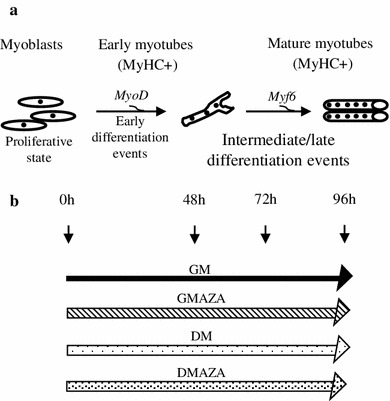
*Up* schematic illustration of skeletal muscle differentiation. *Down* simplified design of experimental procedures

In our differentiation model, early myotubes appeared 48 h after differentiation induction (intermediate phase), and myotubes formation was completed after 72–96 h (late phase). We analyzed DNA demethylation effects, during these stages, adding AZA both in GM and DM cultured cells (GMAZA–DMAZA) at the beginning of the differentiation process. In the first case (GMAZA), we would delineate the possible ability of DNA demethylation to accelerate myoblast spontaneous differentiation due to cell–cell contact; and in the second (DMAZA), we would determinate the probable role of DNA hypomethylation to improve myogenesis process (Fig. 1).

**Fig. 2:**
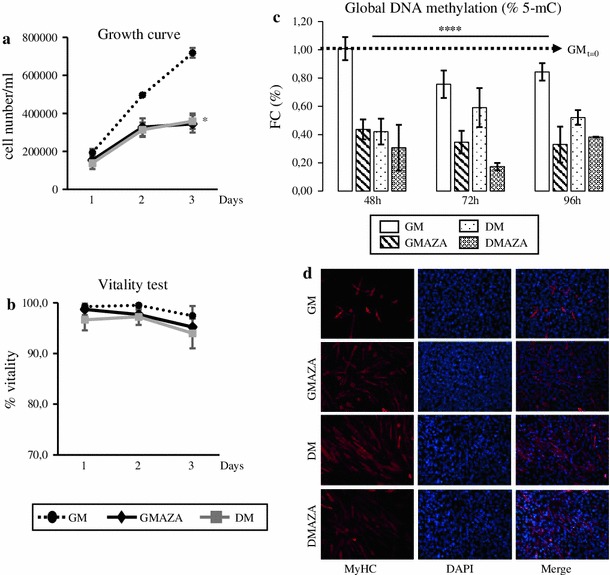
AZA effects on myoblasts proliferation and on global DNA methylation status. **a** Growth curve trends were obtain growing myoblasts until 40 % confluence in GM and then were switched in GMAZA or in DM. The experiment continued until the cells reached subconfluence. GMAZA and DM negative influence C2C12 proliferative potential. **b** Myotoxicity of AZA in vitro: AZA did not induce cell death. **c** Evaluation of AZA demethylation action on global DNA methylation status. Global DNA methylation levels were evaluated by an ELISA assay specific for 5-mC. **d** Immunofluorescence MyHC analysis at 48 h in GM, GMAZA, DM, and DMAZA conditions. MyHC positive cells are labeled in* red*. DAPI and *merge* images are reported

First, proliferation and cell vitality assays were performed in proliferative myoblasts to verify that 5 μM AZA did not have toxic effect (Fig. 2a, b). The cell growth curves showed that AZA and DM induced a significant reduction in the cell growth rate in relation to the untreated control (Fig. 2a). It is important to emphasize that AZA action did not show a cytotoxic effect in C2C12 cells, as revealed cell vitality assay (Fig. 2b). Moreover, in our previous work, we in detail analyzed AZA effect on myoblasts cell cycle progression: we demonstrated that this DNA methylation inhibitor enhances myogenesis induction modulating expression of checkpoint genes involved in cell cycle progression and arrest, but no genes involved in apoptosis phenomena. In fact, GMAZA cells showed a p53 protein content comparable to that of GM cells [45].

Also, to determine whether AZA produces changes in the methylation patterns, we also studied the global DNA methylation by specifically measuring levels of 5-mC in an ELISA-like microplate-based format. As shown in Fig. 2c, we did not observe significant DNA methylation status changes in cells maintained in GM. In contrast, GMAZA and DMAZA cells were characterized by dramatic global DNA methylation reduction (At 48 h FC: 0.44 ± 0.07 GMAZA vs. GM, at 72 h FC: 0.35 ± 0.08 GMAZA vs. GM, at 96 h FC: 0.33 ± 0.13 GMAZA vs. GM; at 48 h FC: 0.31 ± 0.16 DMAZA vs. GM, at 72 h FC: 0.17 ± 0.03 DMAZA vs. GM, at 96 h FC: 0.38 ± 0.003 DMAZA vs. GM; F = 34.35, *p* ≤ 0.00001 anova test followed by Bonferroni post hoc test). In according to the previous data [33, 40], we noticed a significant DNA hypomethylation status in DM condition (at 48 h FC: 0.42 ± 0.09 DM vs. GM, at 72 h FC: 0.59 ± 0.14 DM vs. GM, at 96 h FC: 0.52 ± 0.05 DM vs. GM; F = 34.35, *p* ≤ 0.00001 anova test followed by Bonferroni post hoc test). In detail, DNA methylation status is similar in DM and GMAZA, suggesting that AZA could improve differentiation process regulating epigenetic mechanisms in analogous manner to DM. Furthermore, exposure to DM and AZA resulted in the most significant DNA methylation decreases, indicated that AZA could strengthen muscle differentiation induced by DM. Immunostaining of C2C12 cells with MyHC antibody at 48 h supports the DNA global methylation result: there was an evident increment in the number of MyHC-positive cells in AZA treated than untreated cells (Fig. 2d). These data confirm DNA demethylation action of AZA and indicating that epigenetics regulation is a crucial aspect of skeletal muscle differentiation.

**Fig. 3:**
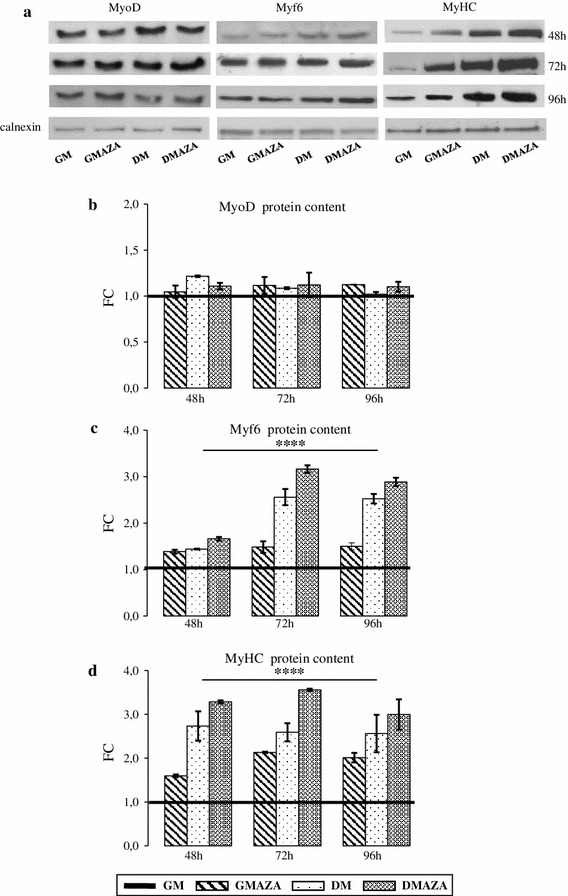
Immunoblotting analysis. **a** Representative immunoblots of analyzed proteins are shown. MyoD (**b**), Myf6 (**c**), and MyHC (**d**) proteins expression was detected by western blot analysis after 48, 72, and 96 h of differentiation induction. C2C12 myoblasts cultured in four different conditions (GM, GMAZA, DM, DMAZA). Data, obtained from three independent experiments, are expressed as fold changes (FC) relative to GM condition. The average, expressed as fold changes (FC) relative to control, are graphically represented. Data were obtained from three independent experiments. Comparisons were performed using one-way analysis of variance (ANOVA) followed by Bonferroni post hoc tests (****P < 0.00001)

For the success of the myogenesis program, sequential expression of MRFs, at specific step, is tightly important [3, 4]. In detail, MyoD reaches the highest expression in early phase and represents a marker for this stage [5, 33]. Instead, Myf6 and skeletal protein MyHC, being expressed during intermediate and late stages of differentiation, are used as markers of these phases [33]. To characterize AZA action on differentiation progression, we performed a detailed temporal study of MyoD, Myf6, and MyHC protein synthesis (Fig. 3a).

Western blot studies showed that MyoD levels were improved (Fig. 3b, FC: 1.22 ± 0.01 DM vs. GM) only in DM cells during intermediate differentiation phase (48 h), according to the previous data [3, 46]. At 48 h, unchanged MyoD expression, observed in AZA treated cells, is maybe due to AZA ability to promote MyoD synthesis. Probably in AZA treated cells, MyoD peak synthesis occurred in the hours before and MyoD protein content is returned to baseline levels at 48 h. In fact, at 72 and 96 h, no significant differences in MyoD content was established between AZA treated and control cells.

As shown in Fig. 3c, at 48 h AZA considerably raise Myf6 protein content and in DMAZA cells this effect was detectable until the late stage (At 48 h FC: 1.66 ± 0.03 DMAZA vs. GM, at 72 h FC: 3.16 ± 0.08 DMAZA vs. GM, at 96 h FC: 2.89 ± 0.09 DMAZA vs. GM; F = 19.41, *p* ≤ 0.00001 anova test followed by Bonferroni post hoc test). Finally, we investigated MyHC protein profile (Fig. 3d). At 48 h, both in GMAZA and DMAZA MyHC content was already higher compared with control (Fig. 3d, FC: 1.60 ± 0.03 GMAZA vs. GM, FC: 3.28 ± 0.03 DMAZA vs. GM, F = 94.05, *p* ≤ 0.00001 anova test followed by Bonferroni post hoc test). In GMAZA, extent of MyHC increase was less than in DMAZA but was prolonged in time (at 72 h FC: 1.49 ± 0.12 GMAZA vs. GM, at 96 h FC: 2.01 ± 0.11 GMAZA vs. GM; F = 94.05, *p* ≤ 0.00001 anova test followed by Bonferroni post hoc test). As expected, in DM condition MyHC amount increased only at 72 h (Fig. 3d FC: 2.56 ± 0.17 DM vs. GM).

These results suggest that AZA has a positive role in MyHC expression and, in association with DM, this effectiveness AZA action is accelerated.

To verify the importance of DNA demethylation during differentiation, we performed morphological analysis, immunostaining C2C12 cells with MyHC antibody.

**Fig. 4:**
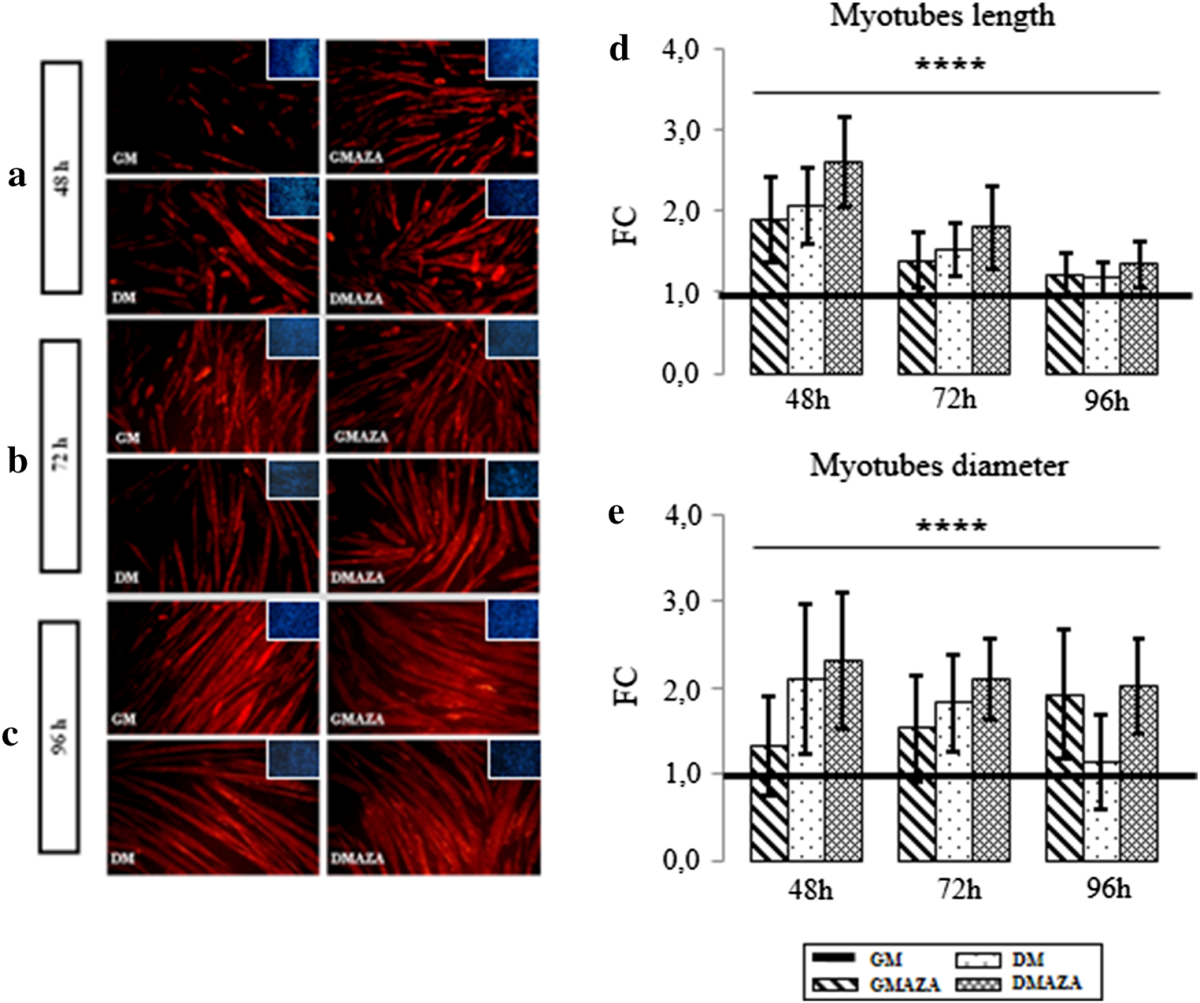
Myotubes immunofluorescence. Analysis and morphological study. MyHC immunofluorescence analysis of C2C12 cells, differentiated for 48 (**a**), 72 (**b**), and 96 (**c**) hours in GM, GMAZA, DM, and DMAZA conditions. MyHC positive cells are labeled in *red*. Representative DAPI images are reported. The length (**d**) and diameter (**e**) of MyHC positive myotubes were measured. The average, expressed as fold changes (FC) relative to GM control, are graphically represented. Data were obtained from three independent experiments. Comparisons were performed using one-way analysis of variance (ANOVA) followed by Bonferroni post hoc tests (****P < 0.00001)

At 48 h (Fig. 4a) in GM condition, MyHC positive cells had small dimensions and shape slightly elongated. GMAZA cells were more elongated, assumed a bipolar morphology and started to orient themselves in the same direction. In DM cells, these processes were more advanced and some groups of cells started to fuse. In DMAZA, this aggregation state was more evident and numerous myotubes were formed. At 72 h, GM cells fused into clusters, while in DMAZA condition organized myotubes were observed. GMAZA and DM cells were in an intermediate condition and showed morphology very similar to each other (Fig. 4b). At 96 h (Fig. 4c), already in GM condition, neo formed myotubes were observed. GMAZA and DM cells had similar morphology, suggesting that AZA action on C2C12 myoblasts is superimposable to DM effect. DMAZA myotubes appeared hypertrophic and these morphological features suggest that DNA hypomethylation could improve myotube formation efficiency.

To support AZA involvement in muscle hypertrophy, myotubes dimensions were measured. During all phases, GMAZA and DMAZA myotubes showed a significant increase in diameter and length compared to GM (Fig. 4, at 48 h diameter FC: 1.33 ± 0.58 GMAZA vs. GM, 2.32 ± 0.79 DMAZA vs. GM; at 48 h length FC: 1.90 ± 0.52 GMAZA vs. GM, 2.60 ± 0.56 DMAZA vs. GM; at 72 h diameter FC: 1.53 ± 0.61 GMAZA vs. GM, 2.10 ± 0.47 DMAZA vs. GM; at 72 h length FC: 1.40 ± 0.34 GMAZA vs. GM, 1.80 ± 0.52 DMAZA vs. GM; at 96 h diameter FC: 1.93 ± 0.75 GMAZA vs. GM, 2.03 ± 0.54 DMAZA vs. GM; at 96 h length FC: 1.21 ± 0.27 GMAZA vs. GM, 1.34 ± 0.28 DMAZA vs. GM; for length F = 17.61, *p* ≤ 0.00001, for diameter F = 27.24, *p* ≤ 0.00001 anova test followed by Bonferroni post hoc test). Moreover, at 96 h, there was still a significant difference between the diameter of GMAZA myotubes and those kept in GM, which instead disappears between the diameters of myotubes grown in DM compared to GM, suggesting a hypertrophic effect of AZA.

**Fig. 5:**
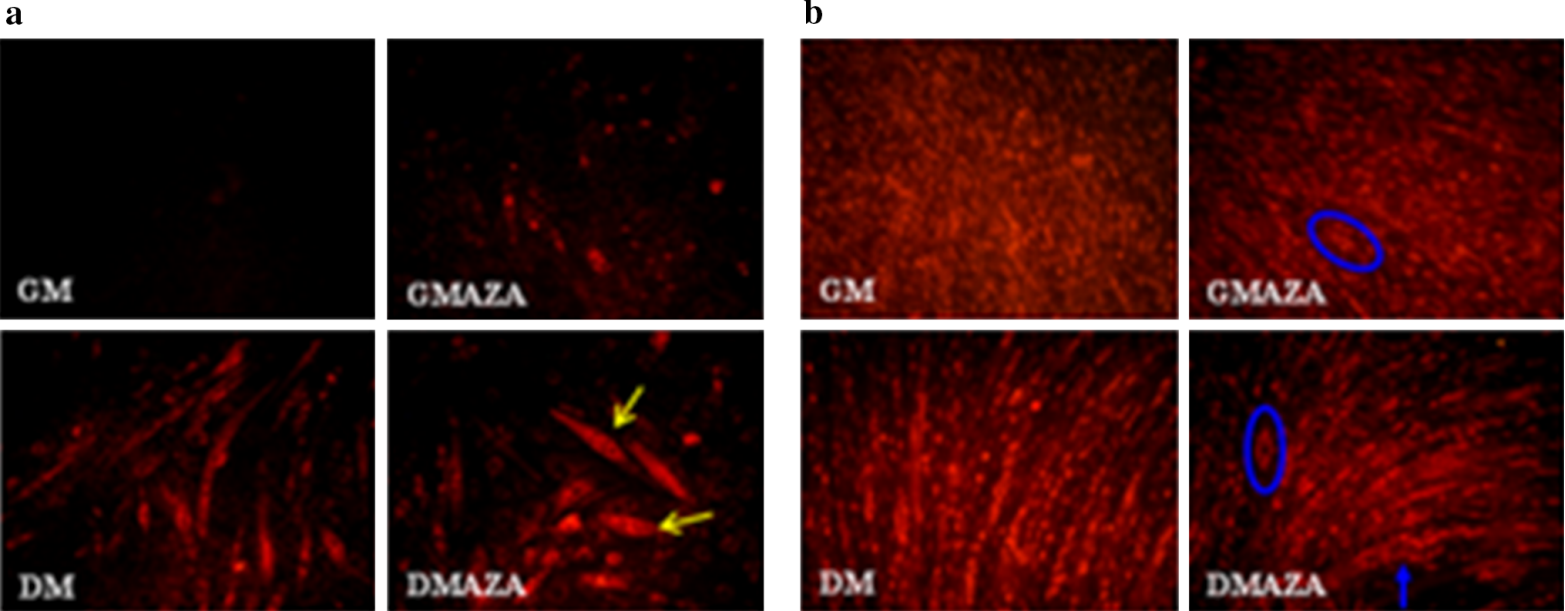
Hypertrophic myotubes morphological study. p21 (**a**) and Mnst (**b**) immufluorescence analysis of C2C12 cells cultured in GM, GMAZA, DM, and DMAZA conditions after 96 h of differentiation. p21 and Mnst positive cells are labeled in *red*

Furthermore, we studied myotubes nuclear disposition during late phase of myogenic differentiation (96 h). As shown in Fig. 5, immunofluorescence analysis, using antibodies against p21 and Myostatin, revealed that AZA treated myotubes were characterized by a particular arrangement of nuclei to form a ring. This nuclear organization represents a morphological marker of in vitro muscle hypertrophy [47].

**Fig. 6:**
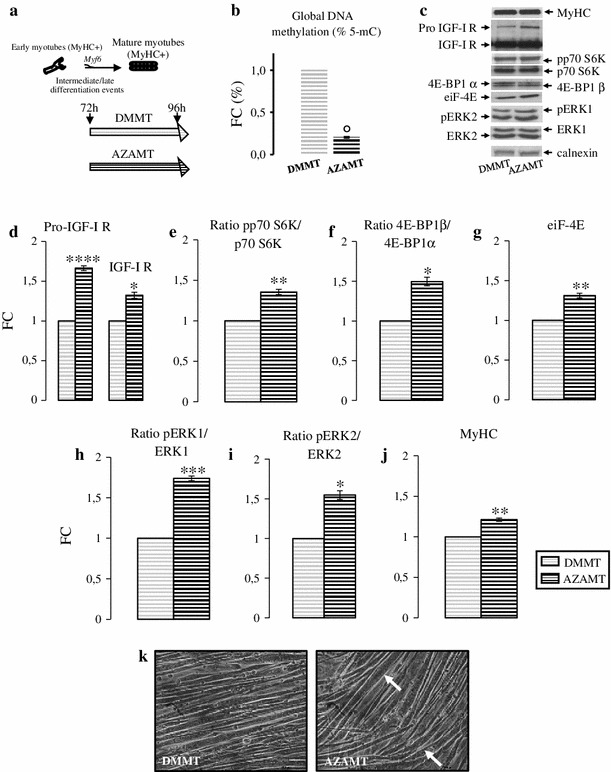
Insulin growth factor I signaling pathway study. **a** Simplified scheme of experimental protocol: C2C12 cells, after 72 h of differentiation induction, were treated for 24 h with (AZAMT) or without (DMMT) AZA. **b** Evaluation of AZA demethylation action on global DNA methylation status. Global DNA methylation levels were evaluated by an ELISA assay specific for 5-mC. **c** Representative immunoblots of analyzed proteins are shown: western blot analysis of Pro IGF-I R and IGF-I R (**d**), pp70 S6 K/p70 S6 K ratio (**e**), 4E-BP1β/4E-BP1α ratio (**f**), eiF-4E (**g**), pERK1/ERK1 ratio (**h**), pERK2/ERK2 ratio (**i**), and MyHC (**j**). Images obtained by bright field microscopy at the end of experiment (**k**). Data, obtained from three independent experiments, are expressed as mean ± SD fold changes (FC) relative to DMMT. Significance: *p < 0.05, **p < 0.04, ***p < 0.03, ****p < 0.02, ^ο^ *p* < 0.00001 versus DMMT condition

To confirm DNA demethylation influence in hypertrophic process, we investigated AZA action on signaling pathway involved in hypertrophy using C2C12 neo formed myotubes. C2C12 neo formed myotubes, after 72 h of differentiation, were treated with AZA for 24 h (AZAMT). Control cells were maintained in DM medium (DMMT), Fig. 6a. As expected, 5-mC ELISA assay revealed a significant DNA hypomethylation in AZAMT compared with unstimulated neo myotubes (Fig. 6b, FC: 0.23 ± 0.02 AZAMT vs. DMMT, *p* ≤ 0.01).

Insulin growth factor I represents a crucial factor in skeletal muscle mass regulation and its activity predominantly requires the binding to IGF-I R [10, 24]. IGF-I R is synthesized as a single polypeptide chain (Pro IGF-I R) that is processed to mature receptor. The previous studies suggest that IGF-I promotes muscle hypertrophy by p70 S6 K and 4E-BP1 activation. 4E-BP1 phosphorylation frees eIF-4E promoting the protein synthesis beginning [24, 29, 31].

Pro IGF-I R protein level was higher in AZA treated myotubes (AZAMT) than in control (DMMT) (Fig. 6d FC: 1.66 ± 0.03 AZAMT vs. DMMT, *p* ≤ 0.02), while AZA action on IGF-1 R expression was less prominent (Fig. 6d, FC: 1.32 ± 0.04 AZAMT vs. DMMT, *p* ≤ 0.05). Probably, deep changes in IGF-I R synthesis could be observed after 96 h of myotubes differentiation.

As expected, AZA treated myotubes were associated with enhanced activation of p70 S6 K, as shown by the increase of the ratio between phosphorylated vs. un-phosphorylated protein (Fig. 6e, p70 S6 K/p70 S6 K FC: 1.36 ± 0.03 AZAMT vs. DMMT, *p* ≤ 0.04).

4E-BP1 has three electrophoretic forms, designated α, β, and γ in order of increasing mobility, inversely related to their phosphorylation state [31]. In our myotubes, 4E-BP1 was present only in the α and β forms. AZA stimuli decreased the amount of α form and increased β form content, as evidenced by the two isoforms ratio (4E-BP1β/4E-BP1α) (Fig. 6f, FC: 1.50 ± 0.06 AZAMT vs. DMMT, *p* ≤ 0.05). Moreover, we observed an eIF-4E protein level increment in AZA neo formed myotubes (Fig. 6g, FC: 1.31 ± 0.03 AZAMT vs. DMMT, *p* ≤ 0.04).

Insulin growth factor I signaling pathway actives the MAPK pathway. ERK1 and ERK2, principal members of MAPK cascade, play a key role in hypertrophic process [10, 26]. We studied ERK1 and ERK2 activation: AZA treatment increased the ratio between phosphorylated versus un-phosphorylated ERK1 and ERK2 (Fig. 6h, i, pERK1/ERK1 FC: 1.74 ± 0.02 AZAMT vs. DMMT, *p* ≤ 0.04, Fig. 5i, pERK2/ERK2 FC: 1.55 ± 0.05 AZAMT vs. DMMT, *p* ≤ 0.05).

Furthermore, Fig. 6j showed an increased tendency in MyHC protein amount in AZA treated myotubes, suggesting an activation of the protein translational machinery (FC: 1.21 ± 0.02 AZAMT vs. DMMT, *p* ≤ 0.00001) and the signaling cascades involved in hypertrophic mechanisms. Finally, to confirm this deduction bright field microscopy images (Fig. 6k) indicated that in AZAMT cell the hypertrophic process was more marked.

## Discussion

Several investigators [34, 40, 45, 48], including us, have shown that DNA demethylation promote MRFs expression and skeletal muscle differentiation induction. In the present study we explored AZA treatment effects during myoblasts differentiation. We, also, investigated AZA action on neo formed myotubes to evaluate its possible impact on hypertrophic process.

Our results demonstrated that during intermediate and late differentiation phase AZA strengthens and promotes Myf6 and MyHC expression compared to untreated cells. The morphological analysis confirmed that myoblasts aggregation, orientation, and fusion come earlier in AZA treated cells. Also, AZA stimuli increases myotubes length and diameter. Therefore, AZA demethylating action shortens the differentiation process, maintaining the ordered and sequential expression of the muscle-specific gene program.

The present in vitro data lead to a number of considerations.

First, our findings point to the interactions between AZA and DM on muscle cells differentiation. Specifically, the AZA–DM association has a synergistic action in enhancing myogenic process and giving hypertrophic stimuli. In fact, only in DMAZA condition p21 and Mnst-positive myotubes showed the nuclear distribution shaped a ring, typical of muscle hypertrophy in vitro [47].

Second, we investigated AZA action on IGF-I signaling pathway involved in skeletal muscle hypertrophy. We observed that AZA-induced DNA hypomethylation could improve IGF-I R protein synthesis. This is supported by a recent study demonstrating that IGF-I R synthesis is regulated by DNA methylation [49]. We evaluated the AZA action on p70 S6 K, 4E-BP1, and eIF-4E proteins, essential regulators of protein synthesis. We demonstrated that AZA activates these proteins that, at the end, lead to a raise of MyHC protein content, reflecting the increased overall protein synthesis. These results suggest the possible correlation between epigenetic mechanisms and muscle protein accretion.

Importantly, our data may have a potential impact in vivo regulation of protein metabolism. In fact, IGF-I is a fundamental mediator of the hormonal and metabolic action of Growth Hormone. GH is secreted in many physiological and stress-related conditions [50]. Our results suggest that AZA might be synergistic to GH in fostering the IGF-I mediated actions [51]. We may speculate an hypothetical clinical use of demethylating agents in conditions of muscle mass impairment/hypotrophy. A synergistic effect of 5-azacytidine and hGH-V (human placental growth hormone) was previously established [52].

Third, the demonstration that AZA-induced DNA demethylation accelerates the skeletal muscle differentiation might constitute a new model to study the patho-physiology of several genetic muscle diseases. Recent studies have evidenced that DNA hypermethylation is correlated with genetic skeletal muscle diseases, like Duchenne muscular and myotonic dystrophy [53, 54]. Hupkes et al. [40] have reported that AZA induced-DNA demethylation enhances skeletal muscle function, in particular AZA promotes the acquisition of spontaneous contractility in C2C12 cell model. Accordingly and concordant with these data, our results demonstrate that DNA hypomethylation could improve skeletal muscle differentiation. Taken together, these data confirm the idea that the DNA methylation could represents the epigenetic key point regulating skeletal muscle differentiation and that drugs or other stimuli, which promote DNA demethylation, could modulate muscle differentiation.

Fourth, our observation that AZA and DM association induces hypertrophic response implies a link with the most important physiological stimulus leading to muscle hypertrophy: exercise training. Very recently, Barrès et al. [55] demonstrated that acute exercise leads to transient changes in DNA methylation in key genes of skeletal muscle metabolism. In addition, our previous study that showed a significant association between elité athletes phenotype (enhanced muscle mass) and polymorphic variants involved in DNA methylation [48]. Considering all together, the above considerations suggest an important regulatory role of DNA methylation on muscle mass accretion.

In conclusion, we demonstrated that DNA demethylation enhances myoblasts differentiation and hypertrophy during the late phase of myogenesis and that this effect is mediated via activation of the IGF-I receptor and post-receptor phosphorylation cascade. The present results constitute a proof of principle of potential effects of 5-azacytidine on muscle protein accretion.
